# Examining Speech Perception–Production Relationships Through Tone Perception and Production Learning Among Indonesian Learners of Mandarin †

**DOI:** 10.3390/brainsci15070671

**Published:** 2025-06-22

**Authors:** Keith K. W. Leung, Yu-An Lu, Yue Wang

**Affiliations:** 1 Department of Linguistics, Simon Fraser University, 8888 University Drive, Burnaby, BC V5A 1S6, Canada; leungkkw@gmail.com; 2 Department of Foreign Languages and Literatures, National Yang Ming Chiao Tung University, Hsinchu 300093, Taiwan; yuanlu@nycu.edu.tw

**Keywords:** speech perception, speech production, perception–production link, second-language learning, Mandarin tones

## Abstract

Background: A transfer of learning effects across speech perception and production is evident in second-language (L2)-learning research, suggesting that perception and production are closely linked in L2 speech learning. However, underlying factors, such as the phonetic cue weightings given to acoustic features, of the relationship between perception and production improvements are less explored. To address this research gap, the current study explored the effects of Mandarin tone learning on the production and perception of critical (pitch direction) and non-critical (pitch height) perceptual cues. Methods: This study tracked the Mandarin learning effects of Indonesian adult learners over a four-to-six-week learning period. Results: We found that perception and production gains in Mandarin L2 learning concurrently occurred with the critical pitch direction cue, F0 slope. The non-critical pitch height cue, F0 mean, only displayed a production gain. Conclusions: The results indicate the role of critical perceptual cues in relating tone perception and production in general, and in the transfer of learning effects across the two domains for L2 learning. These results demonstrate the transfer of the ability to perceive phonological contrasts using critical phonetic information to the production domain based on the same cue weighting, suggesting interconnected encoding and decoding processes in L2 speech learning.

## 1. Introduction

From a theoretical perspective, the perception and production of speech sounds should be closely related. As posited by the gestural theories of speech perception, such as the Motor Theory [1,2,3,4] and Direct Realism [5,6], the two domains are governed by a unitary system, and therefore, should be tightly linked. On the other hand, perception and production are highly synchronized, as predicted by the auditory approaches to speech perception [7,8,9,10], through the feedforward and feedback system in speech perception and production acquisition [11,12,13]. In the same vein, a perception–production relationship should also be found in L2 acquisition, as predicted by the Speech Learning Model [14,15,16]. This has been demonstrated by a transfer of learning effects across perception and production in a number of empirical studies.

### 1.1. Transfer of Learning Effects Across Domains

As shown in laboratory training studies, training individuals to perceive or produce speech sounds of an L2 language improved both perception and production [17,18,19,20,21,22,23,24,25,26,27]. The transfer of learning effects across domains is an indication of a link between perception and production. In addition, the two domains display a semi-autonomous nature. While perception-only training studies unequivocally demonstrated a transfer from perception training to production [17,19,20,21,23,25,26,27,28], this transfer was less than perfect since perception and production gains lacked a correlation [17,28]. On the other hand, production training demonstrated a transfer from production to perception only in some studies [22,24] but not in others [21,29,30]. Moreover, production training could disrupt perceptual learning if the production task involved the sounds to be learned and took place immediately after perception [31,32,33].

While these studies revealed a link between perception and production as semi-autonomous systems for L2 individuals learning a new language, the perception and production improvements were measured by accuracy rates of perceiving and producing sound categories in a number of studies. Specifically, the perception of speech categories was assessed by discrimination or category identification accuracy, whereas production accuracy was determined by the judgment of L2 productions, as evaluated by first-language (L1) listeners of the language of interest, usually through an identification task. However, there have been very few L2 studies that have examined learning effects in both perception and production in terms of particular acoustic cues.

In fact, examining the learning effects through the perception and production of acoustic cues is crucial. First, the theoretical predictions of a perception–production link imply that there is a link between cue weightings in perception and production [34]. However, the aforementioned L2 studies regarding the transfer of learning effects did not clearly establish such a perception–production link on the acoustic cue level, as the perception and production tasks did not measure cue weightings. While some learning studies examined the acoustic properties of L2 productions by investigating the acoustic distance between the productions of L2 sound categories, the perception performance was measured by the perception of actual L2 words [18,26,27]. When a manipulation of acoustic cues was performed to produce perception stimuli, multiple cues were manipulated simultaneously, such that the influence of an individual cue on perception could not be determined [32]. On the other hand, when encountering different speakers, listeners can adjust their relative cue reliance in perception [35]. Therefore, the L1 judges who rate L2 productions may use a cue-weighting pattern that differs from their L1 cue reliance during a rating task, which complicates determining the cue weightings in L2 productions.

More importantly, among the studies comparing cue weightings across the two domains, the findings revealed an asymmetry in L2 perception and production [34]. For instance, previous studies compared the use of fundamental frequency (F0) and voice onset time (VOT) in the perception and production of English voicing contrast by L1 Korean speakers of different dialectal backgrounds [36] or English proficiency levels [37]. Both studies found no difference in cue weightings in L2 production, although some L2 individuals showed a stronger cue weighting on F0 in perception. Similarly, the comparison of L2 perception and production of English vowel contrasts based on the cue weightings given to duration and spectral centroids showed no difference in the use of these cues in production among L1 Spanish individuals varying in the time of onset of their English learning. However, the reliance on duration in perception differed, with late learners depending more on duration than early learners [38]. These results seem to suggest that L2 perception and production lack a relationship in cue weightings. However, it is worth noting that the F0 and duration cues examined in these studies are not critical for the L1 perception of the English sounds being studied. Perceptual cues are considered critical when they are independently informative for phonological perception and production [34]. They influence individuals’ between-category perception of speech sounds, and there is evidence showing that they modulate within-category perception in the rating of speech sounds’ categorical goodness [39]. In contrast, non-critical perceptual cues do not influence the perception of phonological categories on their own. They may only serve as contextualizing cues that modulate the use of critical cues in phonological perception [34,39]. As for the aforementioned L2 studies [36,37,38], F0 and duration cues are used for perceiving the same speech sounds (word-initial stop voicing, vowels) of the L2 English learners’ L1 language (Korean, Spanish). However, unlike critical cues such as VOT for English stop voicing contrasts that are informative on their own in phonological perception for L1 English individuals, F0 and duration do not influence the phonological perception of English word-initial stop voicing and vowels, respectively [34]. Therefore, F0 and duration are considered non-critical perceptual cues, which did not demonstrate a perception and production link for L2 in previous studies [36,37,38].

Instead of non-critical perceptual cues, it is possible that a perception–production relationship for L2 is established through critical perceptual cues. Our recent studies demonstrated that critical perceptual cues are pertinent to establishing a link between L1 perception and production [39,40]. These studies used Mandarin, a lexical tone language, as a test case. Mandarin has four tone categories differing in pitch height and contour: high-level (tone 1), mid-high-rising (tone 2), mid-low-dipping (tone 3), and high-falling (tone 4) [41]. Each Mandarin syllable carries a tone and the lexical meaning of the syllable changes if the tone varies. For instance, /ma/ carrying each of the four tones means “mother” (/ma1/), “hemp” (/ma2/), “horse” (/ma3/), and “to scold” (/ma4/). Our studies revealed that F0 slope, the critical cue for L1 Mandarin tone perception, influenced tone categorization and goodness ratings of Mandarin tone perception more strongly than F0 mean, the non-critical cue.

Specifically, Leung and Wang [39] asked L1 Mandarin individuals to identify the tone categories of resynthesized tones varying in F0 slope and F0 mean orthogonally. The same group of participants produced the four Mandarin tones, and the F0 slope and F0 mean of these produced tones were obtained. Through a bi-directional statistical learning approach, models were trained using F0 mean or F0 slope of either the perceptual responses or the produced tones. The models were then used to classify the tones from the other domain (i.e., the produced tones or the perceptual stimuli). The results showed that both the perception and production models performed well in classification when F0 slope was used as a factor, whereas the model with F0 mean as a factor performed poorly.

These results suggest that F0 slope, as a critical perceptual cue, provides information for perceiving phonological contrasts. Subsequently, a cross-domain, bi-directional statistical modeling showed that only F0 slope could perform well when the perception model was used to classify production data, and vice versa. Therefore, perception and production were found to be related through F0 slope, whereas F0 mean did not show a perception–production link. In addition, although F0 mean was not found to contribute to L1 Mandarin perception, this non-critical perceptual cue contributed to the classification of tone productions. Although these results were obtained from L1 perception and production, L1 and L2 speakers share the same, or similar, speech development trajectories. In particular, the auditory approaches to speech perception [7,8,9,10] and the L2 Speech Learning Model [14,15,16] share similar views on the development of perception and production, and consequently, influence the development of a perception–production relationship. Therefore, an L2 perception–production link should also be established through critical perceptual cues. So far, little research has directly examined whether these cues display a perception–production link among L2 learners.

### 1.2. Learning Effect on Perceptual Cue Weighting

Though exploring whether critical perceptual cues could establish a perception–production link has not been well studied in L2, previous research has shown that perceptual cue reliance can be redirected through laboratory training and classroom learning, such that learners give stronger weightings to the critical perceptual cues for the L2 that they are acquiring. L2 listeners’ perceptual cue weightings could be modified for consonants [42], vowels [43] and tones [44,45,46]. Furthermore, these studies showed that trainees developed a more L1-like cue-weighting pattern. In other words, they weighted the primary and critical perceptual cues of the target language more strongly after training, and consequently, the new perceptual weightings facilitated their perception of L2 speech sounds. Relating to tone perception, Chandrasekaran et al. [44] showed that Mandarin tone training increased L1 English listeners’ perceptual weighting on F0 slope, the critical cue for Mandarin tone perception. Similarly, in Francis et al. [45], Cantonese tone training led L1 Mandarin listeners to increase the weight given to pitch height while decreasing the weight given to pitch direction, resulting in a cue-weighting pattern more akin to that of L1 Cantonese speakers. The modification of cue weighting is also influenced by the interaction between the sound systems of the trainee’s L1 and L2 languages, as Francis et al. [45] showed that L1 English listeners who already gave a strong weighting to F0 height further increased the weighting given to this cue after Cantonese tone training. Since Cantonese has a three-way tone height distinction, the increase in weighting given to F0 height for both L1 Mandarin and English listeners has been attributed to a more fine-grained height distinction in Cantonese tones than in English intonations.

Regarding secondary, non-critical perceptual cues, L2 listeners tend to retain the use of these cues during the acquisition of L2 sound categories, especially when perceptual assimilation occurs. For instance, L1 Japanese listeners perceptually assimilate English /ɹ/ and /l/ sounds to the Japanese /ɾ/ category and show a bias towards the use of non-critical cues (e.g., F2 frequency, consonant–vowel transition duration), rather than the critical F3 cue, for English /ɹ/ and /l/ perception. The weightings of the non-critical perceptual cues were retained in the perceptual learning of English /ɹ/ and /l/, except when stimuli were perceived to be dissimilar to the Japanese /ɾ/ category [42]. As for Mandarin tones, previous research also showed that the cue weight given to pitch height, the non-critical cue, did not change as a result of Mandarin tone perceptual training [44,45]. This is not surprising as L1 English individuals demonstrated perceptual assimilation in the perception of Mandarin tones through single-category assimilation, especially for tone 1–3 and tone 1–4 pairs in the Statement intonation category [47,48].

Apart from laboratory training, the modification of cue weightings has also been shown in a naturalistic classroom learning setting. For instance, Mandarin learners whose L1 was English were found to attain more L1-like cue weighting after the first three months of Mandarin classroom learning. Specifically, Wiener [46] found that in the first three months of Mandarin learning, the weighting given to pitch direction by learners had increased, while the weighting given to pitch height remained unchanged. These results corroborate the findings of the training studies reviewed above. However, it should be noted that Wiener [46] interpreted the perceptual dimensions based on L1 Mandarin listeners’ perception results, even though the distribution of tone categories on the tone space varied with learning duration. For instance, after two months of Mandarin learning, learners used pitch direction to separate tone 1 and tone 4, rather than tone 2 and tone 4. By the third month, learners relied on pitch direction to distinguish tone 2, which has a rising contour, from the other tones. However, tone 4, which has a falling contour, was not correctly positioned at the opposite end of this dimension. As a result, Wiener [46]’s participants displayed a more nuanced development of critical cue weighting. After three months of learning, learners were able to discriminate tone 2 based on pitch direction better than the other tones.

In sum, L2 learning redirects L2 learners to give stronger weightings to the critical acoustic cues that facilitate L2 speech perception [42,43,44,45,46]. In particular, L2 individuals weight pitch direction more strongly after training or three months of learning in Mandarin tone perception [44,46]. It is also possible that a cue-weighting pattern might emerge as an intermediate step, which does not fully resemble the L1 cue-weighting pattern, suggesting different paces of perception development across tone categories for individual learners [46]. Nevertheless, it is still uncertain whether the shift in cue reliance in L2 perception will transfer to L2 production.

### 1.3. The Present Study

The present study explores whether an L2 perception–production relationship can be attributed to the use of critical perceptual cues. To demonstrate this, it investigates the learning effects on perception and production of critical and non-critical perceptual cues. Based on the review above, this study predicts that a transfer of learning effects from perception to production should occur for critical perceptual cues, as these cues play a critical role in establishing a perception–production link. Specifically, the previous training and learning studies suggest that there should be an increase in the weighting given to a critical perceptual cue during L2 learning [42,43,44,45,46]. At the same time, it should also improve the L2 production of the same acoustic cue as a result of a transfer of learning effects [17,18,19,20,21,22,23,24,25,26,27]. As for non-critical perceptual cues (relative to “critical” ones), they should demonstrate a weaker learning effect in perception and should show a weaker transfer of learning effects across perception and production domains.

This study measured the learning effects by tracking the changes in the contribution of F0 slope (critical) and mean (non-critical) to the categorization of Mandarin learners’ perceptual tone identification responses and tone productions in a four-to-six-week Mandarin-learning period. All learners were beginner-level adult Mandarin learners, who had Indonesian as their L1 and English as one of their L2s. In this study, an individual’s L1 refers to the language they first acquired in early childhood, in which they have high proficiency, and which they predominantly use in daily communication. L2 refers to additional languages that an individual acquires later in life, usually in adulthood.

Indonesian, similar to English, is a non-tonal language in which pitch information is used for differentiating pragmatic meanings [49]. Therefore, the L1 Indonesian individuals who were selected as the participant group had no background in any tonal languages except Mandarin. As such, it is expected that, similar to L1 English individuals [45], L1 Indonesian individuals should rely more strongly on F0 height cues, such as F0 mean, in tone perception prior to learning Mandarin. They should also exhibit a lower weighting of F0 slope than L1 Mandarin individuals. On the other hand, these individuals had no existing lexical tone categories to influence their Mandarin tone learning, unlike L1 Cantonese individuals, who tended to categorize Mandarin tone categories based on their L1 Cantonese tone categories during their Mandarin tone learning [50]. As for the acquisition of the Mandarin cue-weighting patterns, it is predicted that the individuals participating in this study should increase their weighting of F0 slope, similar to individuals with English as their L1, which is also a non-tonal language [45], thus allowing this study to observe a change in the reliance on F0 slope through Mandarin learning, and the potential transfer to production.

Following our previous study [39], the cue contributions were examined using a statistical learning method. The cues selected for this study were F0 slope and F0 mean [51,52,53], which have been shown to be the main cues that differentiate Mandarin tones [54]. Although learners may use these Mandarin tone cues differently in their L1, this study aims to track the learning of these cues and achieve the objectives of this study. The perception and production models with F0 slope and/or F0 mean as predictor variables were trained and tested on the data obtained from the same domain, and each model’s performance was assessed by classification accuracy rates. The models were trained on individual learner’s data. As a result, each learner had their own model accuracy for each visit and domain, and consequently, this study was able to compare the improvement in perception and production model accuracy across visits (i.e., learning effect) for F0 slope and mean quantitatively.

Since a simultaneous learning effect in perception and production indicates a link between the two domains, tracking the improvement in perception and production for each cue (i.e., F0 slope and F0 mean) should demonstrate whether an L2 perception–production link is attributed to the critical status of perceptual cues, achieving the aim of this study. Moreover, this design differs from previous training and learning studies which only demonstrated a perception–production transfer predominantly in terms of category identification in perception and L1 speakers’ judgments in production. It also extends previous research, which primarily focuses on changes in cue weighting in perception, to include changes in the use of acoustic cues in both perception and production.

It is expected that the learning effect in perception should be found for F0 slope, as a critical perceptual cue. In contrast, F0 mean, as a non-critical perceptual cue, should show a minimal learning effect. Therefore, the F0 slope model classification accuracy should increase across visits while a significantly lesser degree of increase/change compared to that for F0 slope should be found for the F0 mean model. Based on previous training study results [17,19,20,21,23,25,26,27,28] and the findings in our previous work [39], it is also expected that there should be a transfer of learning effects across the perception and production domains, as demonstrated by a simultaneous increase in the contribution of F0 slope to the classification of Mandarin tone productions in the statistical learning model, if a perception–production link can be attributed to critical perceptual cues. In contrast, the contribution of F0 mean in perception and production should exhibit a less obvious simultaneous change.

It is also worth comparing the perceptual and production gains to investigate whether there is a close connection between perception and production development. As mentioned above, previous studies did not investigate an L2 perception–production relationship at the acoustic cue level [17,28]. Based on the findings of these studies, the two domains display a semi-autonomous nature, and it is conceivable that the relationship between perceptual and production gains is also weak when examining the gains through the use of acoustic cues. Additionally, although previous studies generally found a trend of perception leading production in L2 learning, as demonstrated by greater perceptual gains than production gains [17,28,55,56], there were conflicting findings on the L2 learning of tones. Previous research showed a production lead in Mandarin learning, since learners’ L2 productions had higher accuracy than perceptual identification [57], but also a perception lead, since learners were generally able to accurately perform tone discrimination, while there was much more inter-speaker variability in their productions [58]. Therefore, it is still uncertain which domain should yield greater improvement.

Overall, the novelty of the present study lies in two aspects. First, it explores the L2 perception–production relationship through critical perceptual cues, which extends the current understanding that a transfer of learning effects exists at the category identification level in perception and production [17,19,20,21,23,25,26,27,28]. Second, the present study investigates perceptual and production gains in learning at the acoustic cue level, an aspect not previously explored [17,28].

## 2. Materials and Methods

### 2.1. Participants

The participants were 19 adult learners of Mandarin studying in the Mandarin Chinese program at National Yang Ming Chiao Tung University, Taiwan, between the ages of 19 and 28 (mean: 23.7). The number of learners was comparable to that of previous training and L2 studies (11–21) [17,31,33,44,59,60], some of which used similar statistical approaches, such as linear discriminant analysis and ANOVA (refer to Section 2.4.3 and Section 3 for details about the statistical approaches and analyses). The participants spoke Indonesian as their L1 language (female = 11, male = 8) and did not have any prior knowledge of tone languages other than Mandarin. Mandarin was the third language of the majority of the learners. All learners also spoke English as their second language. In addition, a few learners had knowledge of an additional non-tone language (e.g., Javanese, Arabic). All participants had studied Mandarin courses for 7 months when they were recruited to participate in this study. Based on the program admission interviews, these learners were regarded as beginner-level learners who had minimal knowledge of Mandarin and were still taking beginner-level courses when they participated in this study. They spent 2 to 4 class-hours per week learning Mandarin. The Mandarin classes had a focus on daily verbal communication (i.e., listening and speaking). The teaching objectives were to hold simple daily conversations, such as ordering food, describing locations, activities, and talking about someone’s plans for their free time. In addition, the courses also emphasized learning Chinese characters, phrases, and grammar structures. Therefore, participants developed their phonological knowledge of Mandarin tones through conversations that involved reading and listening to Chinese characters, phrases and sentences, during which they practiced identifying, differentiating, and producing the correct Mandarin tones. The participants estimated that their daily use of Mandarin was between 0% and 24% outside the classroom (mean: 10.3%). They all reported that they used the language in school. Five out of the nineteen learners also used the language with friends and one used it at work.

### 2.2. General Procedures

In order to examine the development of Mandarin perception and production, participants took part in two visits. For each visit, the procedures followed our previous work [39,54]. The production task was always carried out first, followed by the perception experiment. Each participant produced two sets of Mandarin tone data and responded to two sets of perception stimuli. The two visits were 4–6 weeks apart, during which participants continued attending Mandarin classes, engaged in Mandarin tone practice through classroom conversations, and developed their phonological knowledge of Mandarin tones, as mentioned previously. These participants did not engage in any practice similar to the experimental tasks. Therefore, participants should not have become familiar with the experimental tasks when they returned for the second visit. More importantly, the perception and production tasks, as described below, did not involve predefined responses, and no feedback regarding perception or production accuracy was given to the participants. Even if the participants were familiar with the tasks, they should not have been able to adjust their performance to produce any desired outcomes.

### 2.3. Production Task

#### 2.3.1. Materials

The production items were the Mandarin monosyllabic words /ɤ/, /i/ and /u/ (*e*, *yi* and *wu* in *pinyin*) with four Mandarin tones, carrying the meanings “graceful” (/ɤ1/; tone 1), “goose” (/ɤ2/; tone 2), “nauseous” (/ɤ3/; tone 3), “hungry” (/ɤ4/; tone 4), “clothing” (/i1/), “move” (/i2/), “chair” (/i3/), “translate” (/i4/), “dirty” (/u1/), “none” (/u2/), “dance” (/u3/) and “error” (/u4/). Each tone word item was produced in isolation 10 times. Therefore, each participant produced a total of 120 items per visit (3 monosyllables × 4 tones × 10 repetitions).

#### 2.3.2. Procedures

Participants took part in a self-paced production task. In each trial, one word, displayed as both a Chinese character and *pinyin*, was presented in the center of the screen each time. The production items were presented in a random order. They were asked to produce the items clearly, and participants could repeat the production if they made any mistakes. The recording task was conducted in a sound-attenuated booth at National Yang Ming Chiao Tung University.

#### 2.3.3. Acoustic Measurements and Modeling

All productions were analyzed acoustically in Praat (Version 6.0.43) [61]. The tone contour was measured from the beginning and ending of the waveform periodicity of each item to obtain the duration of each tone. The tone contour was divided into 10 intervals of equal distance. F0 values in hertz were then obtained at the 11 equidistant time points along a tone contour. The F0 values were manually checked for accuracy by the first author and phonetically trained research assistants. Any inaccurate or missing F0 data points were manually measured by taking the inverse of the duration of a single period. The data points that were not measurable were removed and treated as missing data (due to aperiodic cycles caused by creaky voice). The removed data points accounted for 1.42% of the total. To normalize for inter-speaker pitch range differences, Equation (1) was used to convert the F0 values to logarithm-based *T* values [27].

(1) $$T=\frac{\log x-\log L}{\log H-\log L}\times 5$$

where *x* is the F0 value in Hz at any given sampling point, and *L* and *H* are the minimum and maximum F0 values, respectively, of all four tones produced by the same speaker. *T* has a range of 0 to 5, mimicking the pitch scale for lexical tones developed in a previous study [41].

To model the F0 mean and slope values of each tone item produced by the participants, the normalized F0 values at the 11 sampling time points were assigned to Equation (2), with normalized time *t* falling in the interval of 0 ⩽ *t* ⩽ 1 (cf. [54]):

(2) $$f\left(t\right)\approx c_{0}+c_{1}\left(t-\frac{1}{2}\right)$$

where $c_{0}$ and $c_{1}$ are the values of F0 mean, the non-critical cue, and slope, the critical cue, respectively. The F0 slope of each tone contour is then divided by the duration of that contour (in s) to obtain a time-scaled slope. A positive and negative F0 slope value indicate a rising and falling contour, respectively.

### 2.4. Perception Experiment

#### 2.4.1. Stimuli

The stimuli were the Mandarin syllable /ɤ/ with superimposed resynthesized and natural tone contours provided by two L1 Mandarin speakers, who were participants in Tupper et al. [54] (1 male, 1 female). The resynthesized tone contours were used to determine the critical status of each perceptual cue, and the natural tone contours served as references. The tone contour resynthesis was carried out by varying F0 mean (7 steps) and F0 slope (11 steps) orthogonally. As a result, the flat contours with F0 slope near 0 correspond to tone 1, particularly those with a high F0 mean. The rising and falling contours reflect tones 2 and 4, respectively. Although these resynthesized tones do not have dipping contours, the falling contours in the mid-low F0 range could resemble the reduced form of tone 3 that appears in connected speech. The parameters of the resynthesized tone contours were based on the speakers’ tone productions. Each speaker produced 98 instances of the Mandarin word /ɤ/ with four tones (22 tone 1 + 24 tone 2 + 30 tone 3 + 22 tone 4). Two phonetically trained L1 Mandarin listeners rated the goodness of each speaker’s production as a member of the intended category on a scale of 1 (poor) to 5 (excellent) in an evaluation task. Tone contours that were creaky or rated below 3 in an evaluation task were excluded. Eventually, 96 productions of the male speaker and 69 productions of the female speaker were retained for the subsequent acoustic modeling to create resynthesized tone contours, using Equation (2).

**Figure 1:**
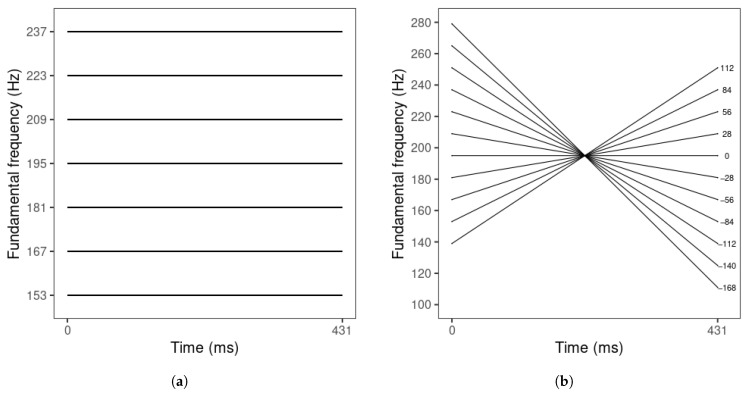
Schematic representations of (**a**) F0 mean series (at F0 slope = 0 Hz/*t*), with F0 mean values displayed on the y-axis; and (**b**) F0 slope series (at F0 mean = 195 Hz), with F0 slope values displayed on the right of each tone.

The endpoints of the resynthesized tone contours’ F0 mean and slope values were based on the mean ranges of F0 mean ($c_{0}$) and slope values ($c_{1}$) across the two speakers. Based on these productions, F0 mean ranged from 153 to 237 Hz. Intermediate steps between the F0 mean endpoints were determined by a step size of 14 Hz, the maximum just-noticeable-difference value for L1 Mandarin listeners [52] (Figure 1a). F0 slope ranged from 112 to −168 Hz/normalized time (*t*). The onset and offset values of the F0 slope endpoints were derived from Equation (2) (at *t* = 0 and 1). Then, the onset and offset were adjusted in opposite directions in 14 Hz steps to obtain the onset and offset values of the intermediate steps. Finally, their slope values were derived from Equation (2) (Figure 1b).

The tone stimuli were resynthesized using PSOLA in Praat [61]. For each speaker, one excellent tone production (rated 5 by both evaluators) was selected. First, the tone duration was modified to 431 ms, the mean tone duration across the two speakers. Then, all the tone sampling points except the onset and offset were removed to create a linear tone contour, and the onset and offset values were adjusted to create flat contours corresponding to the F0 mean values of the seven steps of F0 mean (Figure 1a). After that, for each F0 mean step, the onset and offset were manipulated in opposite directions to create the 11-step F0 slope series (Figure 1). Finally, all tone stimuli were scaled to 70 dB in intensity.

For the natural tone stimuli, two items for each tone were selected from each speaker. All items were free of creak and rated as good productions (goodness ratings above 4) by both evaluators. The tone duration was modified to 431 ms and the intensity was scaled to 70 dB, matching those of the resynthesized tone stimuli. As a result, the perception stimuli consisted of 170 items with resynthesized (7 F0 mean steps × 11 F0 slope steps) and natural tones (4 tones × 2 repetitions) produced by two speakers [(7 F0 mean steps × 11 F0 slope steps + 4 natural tones × 2 repetitions) × 2 speakers].

#### 2.4.2. Procedures

All participants took part in the perception experiments after completing the production task. All stimuli were presented twice (the total number of stimuli was 240). The stimuli were arranged in four blocks. Each block contained one repetition of the stimuli from one speaker.

The experiment involved a four-alternative forced-choice identification. In each trial, one stimulus was presented to the participant, and they were asked to determine the tone category of the stimulus (T1, T2, T3, or T4). The forced-choice identification task aimed to examine how each F0 cue influenced tone categorization. Participants were encouraged to complete each task as quickly as possible, and they had a time limit of 4 s. The natural and resynthesized tone stimuli were mixed and randomized. The block orders were counterbalanced. This experiment was designed with only linear tone contours to independently manipulate one critical cue (F0 slope) and one non-critical cue (F0 mean), for testing the hypothesis of this study. The stimuli, however, did not represent the typical dipping contour of tone 3. The slightly falling tone contours in the mid-low F0 range may resemble the reduced form of tone 3 that appears in connected speech as mentioned above. Therefore, if the learners’ perception resembled that of L1 Mandarin individuals, the number of tone 3 responses would likely be low. However, given that the participants were beginner-level Mandarin learners, it is also possible that some of them were less familiar with the connected speech variant and thus failed to identify these contours as tone 3. Nonetheless, the design enabled us to include all Mandarin tones to examine the learning effects of critical and non-critical cues.

#### 2.4.3. Statistical Approach

Statistical modeling was performed on the perception and production data. For the perception data, a multinomial logistic regression using the nnet package (Version 7.3-17) [62] in R (Version 4.0) [63] was used to explore the influence of F0 mean and slope on tone responses. Each dataset per learner contained at most 308 responses, with either F0 mean (153–237 Hz) or F0 slope (112 to −168 Hz/*t*) as a predictor variable in each model. The dependent variable was tone identification responses (tone 1, tone 2, tone 3, tone 4) at each visit. All models had tone 1 as the reference level. In order to test which variable performed better in tone categorization, the mean deviance statistic (*D*) (minus 2 times the log-likelihood of the model) and mean classification accuracy were obtained using a five-fold cross validation method to avoid overfitting. The data were equally divided into five subsets. Five models were then trained, with four subsets used for training and one subset reserved for testing. Each learner had three models: (1) a null model, containing only the intercept; (2) an F0 slope-only model; and (3) an F0 mean-only model. The differences in *D* between the null and each one-predictor model were used to evaluate model fit qualitatively, and the classification accuracy rates were used to compare between models statistically.

To analyze the influence of F0 slope and mean on production tone categorization, a linear discriminant analysis (LDA) was performed for each learner and visit. An LDA was chosen instead of a multinomial logistic regression because there were fewer observations in each learner’s production data (*n* = 120) than in their perception data (*n* = 308). With a limited number of observations, most learners’ multinomial logistic regression models would fail to converge. However, because there were equal numbers of observations in each tone category, LDA was an appropriate model. The classification accuracy of the models with the linear combination of F0 slope and F0 mean (i.e., F0 slope + F0 mean), F0 slope-only, and F0 mean-only were compared to test which cue had a stronger influence on production tone categorization across visits [54]. The classification accuracy was obtained by the five-fold cross validation method used in the perception data modeling above.

## 3. Results

The goal of this study is to compare the perception and production performance of Mandarin learners across two visits to understand the learning effect on the perception and production of F0 slope and mean. Specifically, the contributions of each acoustic cue (i.e., F0 mean or F0 slope) to tone categorization, assessed by model accuracy, were examined separately in perception and production for each visit. Since learners showed noticeable inter-speaker variability in perception and production, the statistical learning was carried out separately for each learner. That is, each set of a learner’s perception and production data obtained in either visit 1 or visit 2 was used to train and test a statistical learning model that consisted of F0 slope and/or F0 mean as predictor factors. For perception data, each learner’s set of data was analyzed by a multinomial logistic regression model to categorize their perceptual responses using F0 mean or slope alone. The production data were analyzed in the same manner using a linear discriminant analysis. The model classification accuracy was used to indicate the contribution of F0 mean or slope to Mandarin tone perception and production by these learners for each visit. The learning effect was shown by the improvement in model classification accuracy from visit 1 to visit 2. The comparison between the classification accuracy of the F0 slope and mean models illustrated the contribution of each cue to perception and production. A simultaneous improvement across visits for perception and production would indicate a perception–production link. Finally, as a measure of the pace of learning across domains, the correlation between the gains in perception and production was also examined.

### 3.1. Perception Model Classification Results

**Figure 2:**
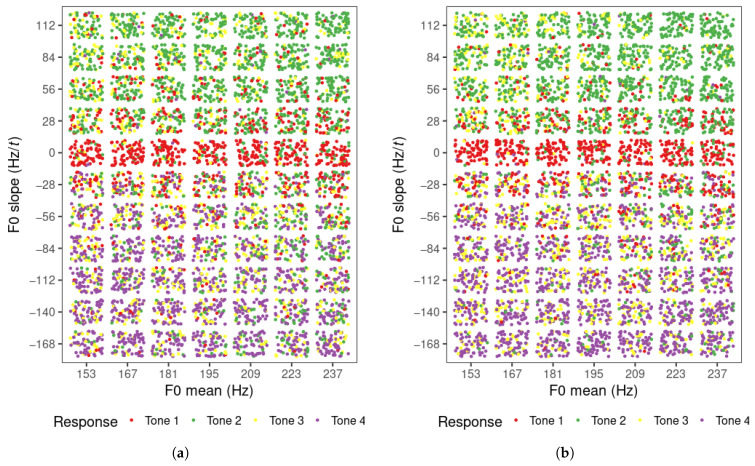
Tone responses of Mandarin learners in perception task for each F0 mean and slope level for (**a**) visit 1 and (**b**) visit 2.

The tone categorization responses of all learners for each level of F0 mean and slope value are displayed in Figure 2a (visit 1) and Figure 2b (visit 2). There were 5852 responses in total per visit (308 per learner = 77 stimuli per block × 4 blocks). The trials in which participants did not give a response were removed (visit 1: 96; visit 2: 30). In general, the majority of the rising, level, and falling tone items were identified as tone 2, tone 1, and tone 4, respectively, and were within the expected range of each of these tones. That is, the contours with positive, near-zero, and negative slope values were mostly identified as tone 2, tone 1 and tone 4, respectively. The tone 3 responses were dispersed, but were found mostly with low F0 mean in the rising tone region and in the falling region, especially in visit 2. It appears that the tone boundaries between tones 1 and 2, and tones 1 and 4 are horizontal on the scatterplots, indicating that F0 slope was the only factor that influenced the perception of tones 1, 2, and 4. F0 mean may only have an impact on tone 3 perception when the tone contour is rising. The distribution of tone responses for these three tones did not appear to differ across the two visits.

**Figure 3:**
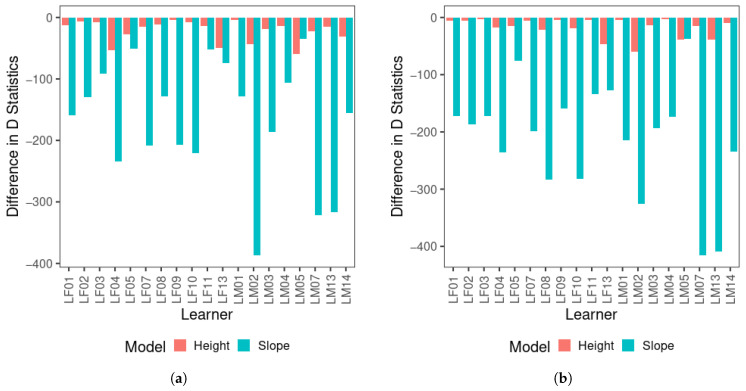
Difference in *D* statistics between the null model and single-predictor models for all learners for (**a**) visit 1 and (**b**) visit 2.

For the multinomial logistic regression results, Figure 3 displays the difference in *D* between the null model and each of the single-predictor models across two visits for all learners (Δ*D*). Note that a smaller *D* represents a better model fit, and therefore, the null model containing only the intercept should have the greatest *D*. A greater drop in *D* compared with the null model, as demonstrated by a longer bar in the figure, indicates that the factor of the model is a better predictor than the factor of the model in comparison with a smaller drop in *D*, i.e., a shorter bar in the figure. As shown in Figure 3, there was a substantial drop in *D* statistics from the null model to the F0 slope-only model, and the drop in *D* statistics for the F0 mean-only model was comparatively limited across visits for most learners. The only exception was LM05, who yielded a greater drop in *D* for F0 mean than F0 slope, but the difference was reduced in visit 2 compared to visit 1. These results indicate that F0 slope improved the model fit considerably compared to F0 mean for the vast majority of learners, and therefore, F0 slope was the critical perceptual cue for tone categorization for them since the first visit. The Δ*D* between the null and F0 slope-only model appears to increase in visit 2 compared to visit 1 for a number of learners.

**Figure 4:**
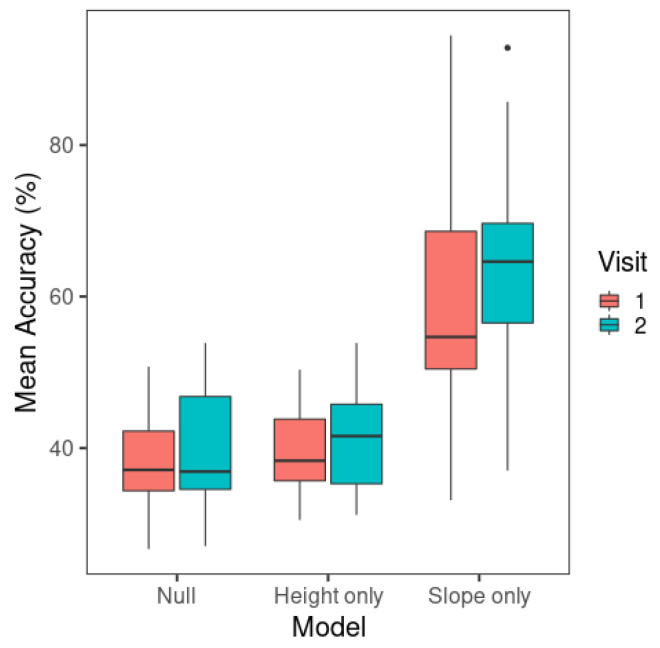
Mean classification accuracy of multinomial logistic regression models across visits. The whiskers of the boxplots represent values within 1.5 times of the interquartile range above the 75th and below the 25th percentiles. A dot represents any value outside that range beyond either end of the whiskers. (Null: Model containing only the intercept; Height only: F0 mean-only; Slope only: F0 slope-only).

Next, to test whether the models displayed a learning effect from visit 1 to visit 2, and whether F0 slope yielded a better performance than F0 mean, the classification accuracy rates of the three models (i.e., null, F0 mean-only, and F0 slope-only) were compared between the two visits. As presented in Figure 4, the F0 slope-only model performed much better than the F0 mean-only model, consistent with the Δ*D* results. The F0 mean-only and null models did not show an observable difference.

These results were further examined in a repeated-measures ANOVA with the afex package (Version 0.28-1) [64]. The independent variables were visit (1, 2) and model (null, F0 mean-only, F0 slope-only). The mean classification accuracy of the multinomial logistic regression models of individual learners was the dependent variable. The tests for violation of the sphericity assumption yielded significant results for model (*W* = 0.279, *p* < 0.001) and the visit × model interaction (*W*= 0.279, *p* = 0.006), so the Greenhouse–Geisser correction was used for these test results. A significant result was detected for the main effect of visit (*F*(1,18) = 6.73, *p* = 0.018) and model (*F*(1.16,20.9) = 48.5, *p* < 0.001). Overall, the models showed a higher mean accuracy in visit 2 (48.3%) than in visit 1 (45.6%). The interaction between visit and model was not significant (*F*(1.38,24.8) = 2.02, *p* = 0.164).

Post hoc pairwise comparisons were conducted for model with Tukey’s adjustment using the emmeans package (Version 1.6.0) [65]. The F0 slope-only model had a significantly higher accuracy (mean = 61.3%) than the F0 mean-only model (mean = 40.6%) (*t*(18) = −6.68, *p* < 0.001) and the null model (mean = 39.1%) (*t*(18) = −7.64, *p* < 0.001). The F0 mean-only and null models did not show a significant difference in model accuracy (*t*(18) = −1.44, *p* = 0.340).

The above results demonstrate that the F0 slope-only model resulted in a significant improvement of model categorization accuracy compared to the null model, but the F0 mean-only model did not perform better than the null model that contained no predictor. Therefore, the categorization performance of the F0 mean-only model simply reflected the tone response that was *a priori* more likely than other tones [66]. Although the ANOVA yielded a significant main effect of visit, the improvement in model accuracy across visits for the F0 mean-only model did not capture an improvement in categorization performance by this acoustic cue. To confirm whether F0 slope alone resulted in a better categorization performance in visit 2 than in visit 1, a separate paired-sample *t* test was carried out with visit as the independent variable and model accuracy as the dependent variable for F0 slope model data only. This yielded a significant difference (*t*(18) = −2.70, *p* = 0.015), indicating that the F0 slope model had higher model accuracy in visit 2 (63.8%) than visit 1 (58.8%), and therefore, there was an increase in the weighting of this cue in perception.

### 3.2. Production Model Classification Results

**Figure 5:**
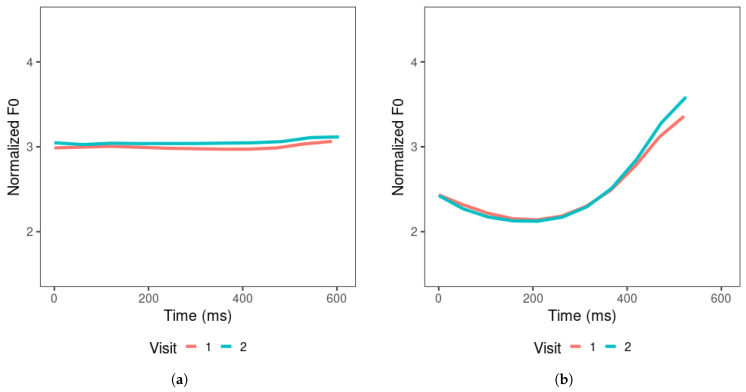

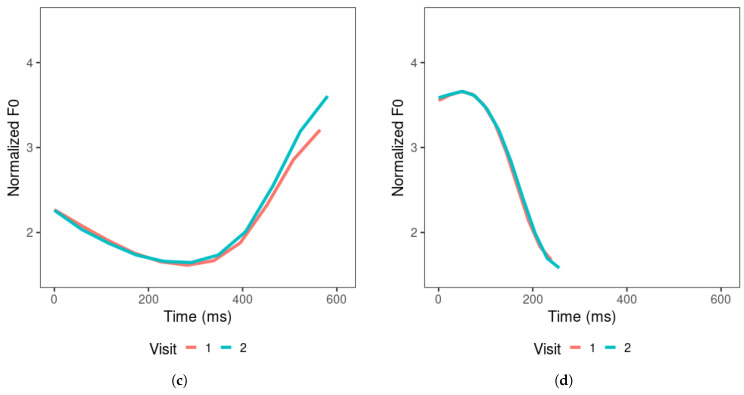
Learners’ productions of (**a**) tone 1, (**b**) tone 2, (**c**) tone 3, and (**d**) tone 4, averaged across items produced in visits 1 and 2.

Figure 5 depicts the mean tone contours for the productions of all learners, which represent the general results of the tone productions across learners and the changes across visits. For productions across visits, the tone contours indicate that learners generally produced higher tone 2 and tone 3 offsets. Tone 4 showed a slightly higher onset and slightly lower offset in visit 2 than in visit 1, suggesting that these tones became steeper as the learners went through the 4–6 weeks of Mandarin learning. The overall F0 mean increased across visits for tones 1, 2, and 3, but remained similar for tone 4 across visits. Learners produced tone 1 with the target-level contour, and on average, the overall F0 mean was slightly higher in visit 2 than in visit 1. Tone 4 was produced with a substantially shorter duration compared to other tones, leading to a steep falling slope.

**Figure 6:**
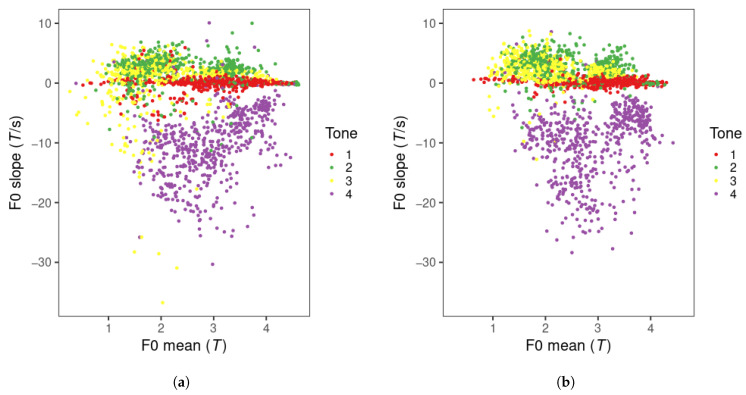
Mandarin tone productions of all learners in F0 mean and slope across visits. (**a**) visit 1; (**b**) visit 2.

The distribution of tone production items in the F0 mean and F0 slope tone space is displayed in Figure 6. For both visits, tone 1, 2, and 4 items are distributed along the F0 slope dimension. Tone 1 occupies the region close to an F0 slope of zero, indicating a flat tone contour. Tones 2 and 4 are found in the positive (rising) and negative (falling) slope regions, respectively. A small number of tone 2 productions appear in the falling slope region. Tone 3 productions are more dispersed in visit 1 than in visit 2 and are mostly found in the rising slope region, overlapping with tone 2 productions in visit 2. Tone 2 appears to have slightly more productions in the high F0 mean region than tone 3.

**Figure 7:**
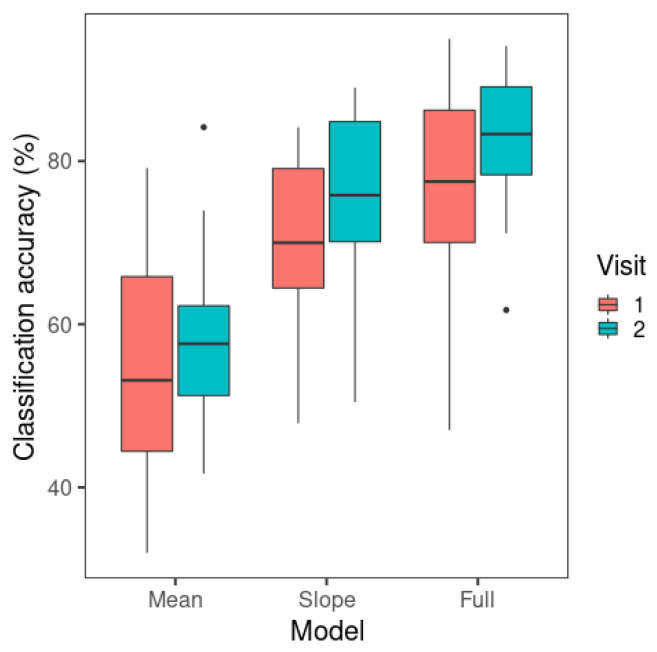
Mean classification accuracy of linear discriminant analysis across visits. The whiskers of the boxplots represent values within 1.5 times of the interquartile range above the 75th and below the 25th percentiles. A dot represents any value outside that range beyond either end of the whiskers. (Mean: F0 mean-only; slope: F0 slope-only; full: F0 slope + F0 mean).

The LDA results are presented in Figure 7. The F0 mean-only model yielded the lowest model classification accuracy among all the models. The classification accuracy of the F0 slope-only model is noticeably higher than that of the F0 mean model but lower than the F0 mean + F0 slope model. The figure displays some increases in classification accuracy from visit 1 to visit 2, especially for the F0 slope-only model.

A repeated-measures ANOVA was carried out with the afex package (Version 0.28-1) [64]. The independent variables were visit (1, 2) and model (F0 mean-only, F0 slope-only, F0 mean + F0 slope). The mean classification accuracy of LDA for individual learners was the dependent variable. Model (*W*= 0.602, *p* < 0.001) showed a significant result in the test of sphericity, so the Greenhouse–Geisser correction was used for the ANOVA results of this effect. Significant main effects were found for visit (*F*(1,18) = 6.71, *p* = 0.018) and model (*F*(1.21,21.7) = 88.3, *p* < 0.001). Overall, there was a higher mean classification accuracy in visit 2 (mean = 74.1%) than in visit 1 (mean = 69.5%). The visit × model interaction was not significant (*F*(2,36) = 2.01, *p* = 0.149).

The significant main effect of model was followed up by post hoc pairwise comparisons with Tukey’s adjustment using the emmeans package (Version 1.6.0) [65]. The F0 mean + F0 slope model had a higher classification accuracy (mean = 79.7%) than the F0 slope-only model (mean = 72.6%) (*t*(18) = −6.06, *p* < 0.001) and the F0 mean-only model (mean = 56.8%) (*t*(18) = −14.8, *p* < 0.001). The F0 slope-only model had a higher classification accuracy than the F0 mean-only model (*t*(18) = −6.69, *p* < 0.001).

The results indicate that learners’ Mandarin tone productions are best categorized using both F0 slope and F0 mean, since the model with F0 slope and F0 mean as predictors showed the highest classification performance. The single-predictor models demonstrated the contribution of F0 slope or F0 mean alone to Mandarin tone productions. As in perception, F0 slope alone was more critical than F0 mean alone in tone production categorization. However, unlike perception model classification, F0 mean showed an above-chance-level (25%) mean classification accuracy, suggesting that this cue alone could also contribute to tone categorization in production.

### 3.3. Correlation Analysis of Perception and Production Gains

**Figure 8:**
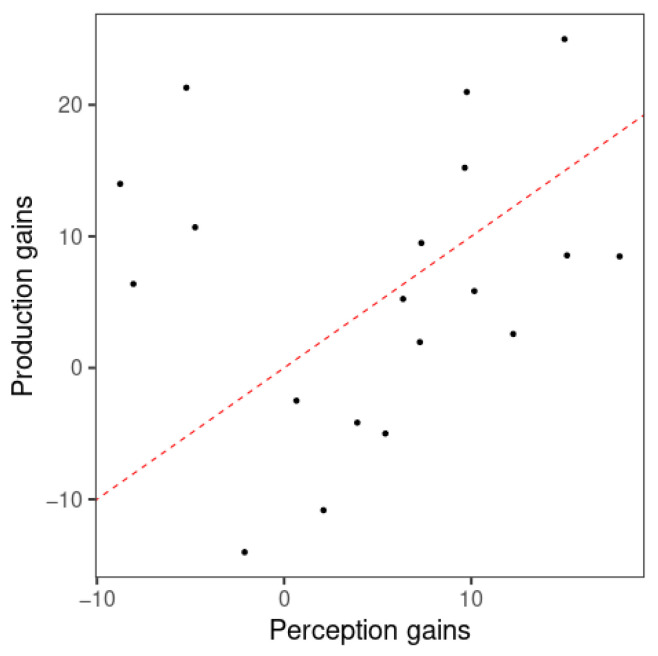
Scatterplot of perception and production gains from visit 1 to visit 2 of the F0 slope model. Each point represents one participant. Red dashed line represents equal amounts of perception and production gains (i.e., y = x).

The relationship between perception and production gains from visit 1 to visit 2 was examined by a correlation analysis of the gains in model classification accuracy of perception and production. Since F0 mean did not show better classification performance than the null model in perception, the gains of the F0 slope-only models alone, obtained from the improvement in classification accuracy per learner, were examined using a Spearman’s rank-order correlation due to a violation of the normal distribution caused by outliers. As expected, no significant correlation was found for F0 slope (ρ(17) = 0.168, *p* = 0.244). As displayed in Figure 8, the data points are found on both sides of the red dashed line representing near-equal amounts of perception and production gains. Therefore, there was not a clear indication of greater gains in either perception or production. While 11 learners showed greater perception gains, 8 learners displayed greater production gains.

## 4. Discussion

This study examined the learning effects of the contribution of F0 mean and F0 slope to perception and production for beginner-level Mandarin learners. The results confirmed the prediction that the F0 slope-only models showed improved classification accuracy from visit 1 to visit 2 for both perception and production. On the other hand, only the production data demonstrated an improvement across visits in classification accuracy for the F0 mean-only model. As in previous studies [17,28], this study did not find a correlation between perception and production gains for overall classification accuracy. The implications of these results on the perception–production relationship in L2 are discussed below, along with a comparison to the relationship between the two domains in L1 languages, referencing our previous work using the same statistical modeling method [39].

### 4.1. L2 Perception–Production Links Established Through F0 Slope, the Critical Perceptual Cue

This study showed that only F0 slope, as a critical perceptual cue, displayed categorization performance that was driven by the cue itself in the multinomial logistic regression analysis in both visits, as the model accuracy was higher than that of the null model. The result indicates that F0 slope played a predominant role in learners’ Mandarin tone perception for perceiving phonological contrasts. The categorization performance is consistent with L1 Mandarin perception explored in our previous work, in which the F0 slope-only model showed much higher classification accuracy than the null model [39], indicating that L1 and L2 Mandarin perception demonstrated a similar cue-weighting pattern. In addition, this study also found an increase in classification accuracy of the F0 slope-only model in perception across the two visits. Therefore, learners increased the weight they gave to F0 slope, the critical perceptual cue, for categorizing Mandarin tones from visit 1 to visit 2, corroborating previous tone perception training and learning studies that found an increase in perceptual weighting on pitch direction [44,45,46] and the training studies that showed increased cue weights given to a critical perceptual cue in a few weeks of exposure to an L2 [23,43].

More importantly, the simultaneous improvement in perception and production of F0 slope extends the findings of previous L2 studies on transfer in learning effects [17,19,20,21,23,25,26,27,28], since it provides further evidence that establishing a perception–production link is attributable to the use of critical perceptual cues [34]. In contrast, the F0 mean-only model showed an improvement in classification accuracy in production only. Therefore, a simultaneous improvement in both perception and production was not found for F0 mean, the non-critical perceptual cue. As noted above (Section 1.1), a transfer of learning effects across the perception and production domains was a factor driving the simultaneous improvement. In addition, learners performed the same tasks in the two visits and the same cues were measured in both perception and production. Therefore, this study directly examined the use of the same F0 slope and F0 mean cues in learners’ perception and production of Mandarin tones. Consequently, the simultaneous learning effect on the F0 slope-only model’s accuracy can clearly be attributed to the acoustic cue itself in both perception and production domains. Note that, in addition to the simultaneous improvement in the perception and production of F0 slope, the study also analyzed the correlation between perception and production gains for F0 slope to further examine whether these parallel improvements occurred at the same rate across L2 learners. However, it did not find sufficient evidence to support this view based on the analysis; for example, a perception lead in L2 learning, as reported in previous studies [17,55,56,58].

It should be noted that, compared to L1 Mandarin perception, the classification accuracy of most learners’ F0 slope-only model was lower than the overall L1 Mandarin individuals’ F0 slope-only model, as revealed in our previous work [39]. This indicates that learners with 7 months of Mandarin learning experience did not rely on F0 slope as heavily as L1 Mandarin individuals in Mandarin tone categorization. Although learners were shifting closer to the L1 direction, as the weighting on F0 slope continued to rise over a period of 4–6 weeks of Mandarin learning, the learners’ F0 slope-only model of visit 2 still attained a lower accuracy rate than that of L1 Mandarin individuals. While the result is not surprising given that the participants were beginner-level learners and the time interval between the two visits was short, learners’ perception of Mandarin tones may involve other cues. Previous studies suggest that a possible cue involved in L2 Mandarin tone perception is pitch height [44,45,51,67]. This study does not fully support this view since F0 mean did not contribute to learners’ Mandarin tone perception. The accuracy of the F0 mean-only model was not different from that of the null model. However, it is possible that learners used pitch height-related cues to perceive Mandarin tones, such as F0 onset [40,53]. Still, their patterns should continue to change as they gain further linguistic experience in Mandarin.

Lastly, this study raises a further question about how the modification of cue weighting will influence the performance of Mandarin learners in real-world, naturalistic Mandarin tone perception and production, which holds important implications for research in L2 learning and Mandarin pedagogy. Although this study showed improvement in learners’ perception and production of F0 slope, this does not necessarily mean that learners categorized the tone items like L1 Mandarin individuals. In production, learners still showed a substantial overlap of tone 3 with the other tones, especially tone 2, in the F0 mean and slope tone space, suggesting challenges in Mandarin tone production. The real-world performance of Mandarin learners should be tested through further studies, such as a perception test using materials from actual L1 Mandarin productions, and an L1 speaker judgment task involving materials produced by L2 speakers.

### 4.2. L2 Learning Effect on F0 Mean, the Non-Critical Perceptual Cue

In perception, the F0 mean-only model did not show any difference in classification accuracy from the null model in both visits, suggesting that F0 mean did not have a clear contribution to the perceptual tone categorization for learners whose L1 language was Indonesian—a non-tonal language. This is consistent with L1 Mandarin perception results, in which no noticeable difference was found between the F0 mean-only and null model [39]. Moreover, the improvement in classification accuracy across visits could not be attributed to the cue itself, since a difference between the classification accuracy of the F0 mean-only and null models was not detected in either visit. Therefore, such improvement did not show that F0 mean became a stronger perceptual cue weighting after 4–6 weeks of Mandarin learning. Together with the F0 slope-only model results, this study showed that Mandarin learners with a non-tonal language background already gave a low perceptual weighting to F0 mean, as in L1 Mandarin perception after 7 months of Mandarin learning.

As for production, the model accuracy of the F0 mean-only models across visits was above chance level, showing that F0 mean had an influence on learners’ tone production. This is also consistent with L1 Mandarin production results, in which F0 mean contributed to the classification of their tone productions [39]. This study showed that the F0 mean + F0 slope models yielded a higher classification accuracy than the F0 slope-only model, further indicating that F0 mean played a role in Mandarin production for L2 speakers. The same results were also found in our previous study with L1 Mandarin individuals [39]. As discussed above, the F0 mean-only model in perception did not show an improvement in tone classification that was due to the use of F0 mean, so only F0 mean-only models in production showed an actual learning effect. Therefore, F0 mean models did not show a simultaneous improvement in perception and production and indicated a lack of transfer of production improvement to perception. This suggests that there was no perception–production relationship through F0 mean, the non-critical perceptual cue, corroborating the findings of previous studies on the L2 perception–production relationship involving acoustic cues [36,37,38]. The learning effect shown only for F0 mean in production suggests that the critical nature of a perceptual cue did not always correspond to the use of the cue in production during the course of Mandarin learning.

The fact that F0 mean played a minimal role in learners’ Mandarin tone perception is not consistent with previous studies that found L1 English individuals to rely on both pitch direction and height in tone perception [44,46] and to show high perceptual weighting on pitch height or F0 mean in tone perception [45,51,67]. This study attributes it to the difference between the results of tone categorization in this study and the tone discrimination results in previous studies that used multi-dimensional scaling (MDS). MDS studies analyze data collected through a discrimination task [44,45,51,67]. In contrast, this study employed a tone categorization task focused on between-category perception. This difference suggests that while both cues may be used in discriminating between four Mandarin tones, F0 mean, as a non-critical perceptual cue, is minimally used in Mandarin tone categorization for beginner-level Mandarin learners.

It should also be noted that the perceptual weight given to the non-critical cue was predicted to remain unchanged across the two visits based on previous training studies [42,44,45], which implies that F0 mean had very little influence on these learners’ Mandarin tone perception at the beginning of their exposure to Mandarin. This seems to suggest that there is a possible cross-linguistic difference between the tone perception of non-tonal language speakers, since L1 English individuals with no Mandarin experience were found to weight pitch height strongly [51,67]. However, another possibility is that, contrary to the prediction, the use of F0 mean in tone perception diminished in the first 7 months of Mandarin learning for the learners of this study. This speculation is possible because the learners who participated in this study had already studied Mandarin for 7 months. In contrast, previous studies only trained participants to learn an L2 language, without any knowledge of the target language, for a few weeks [42,44,45]. Therefore, it is possible that the weighting given to pitch height decreased over the prolonged learning period. This speculation needs to be further examined in a longitudinal study covering a longer L2 learning period from the beginning of individuals’ L2 learning.

## 5. Conclusions

This study demonstrated that an L2 perception–production relationship can be attributed to the use of critical perceptual cues. By comparing the perception and production of Mandarin learners, this study revealed that L2 individuals increased their weighting of the critical perceptual cue, F0 slope, when perceiving resynthesized linear tone contours as Mandarin tones, suggesting that they rely more strongly on critical perceptual cues when perceiving L2 phonological contrasts during L2 acquisition. A simultaneous improvement was found in using F0 slope to categorize their Mandarin tone productions, indicating a connection between the perception and production domains through critical perceptual cues for L2 learners. This connection was not found for the non-critical perceptual cue, F0 mean, as improvement in the use of this cue was observed in production only. All in all, as the use of critical perceptual cues relates to the ability to perceptually encode and decode speech sounds of a language, the findings of this study suggest that harnessing this ability is helpful for improving both L2 perception and production.

## Data Availability

The data presented in this study are available on request from the corresponding author due to privacy concerns.
